# A Web-Based Study of HIV Prevention in the Era of Pre-Exposure Prophylaxis Among Vulnerable HIV-Negative Gay and Bisexual Men, Transmen, and Transwomen Who Have Sex With Men: Protocol for an Observational Cohort Study

**DOI:** 10.2196/13715

**Published:** 2019-09-17

**Authors:** Denis Nash, Matthew Stief, Caitlin MacCrate, Chloe Mirzayi, Viraj V Patel, Donald Hoover, David W Pantalone, Sarit Golub, Gregorio Millett, Alexa B D’Angelo, Drew Anne Westmoreland, Christian Grov

**Affiliations:** 1 Institute for Implementation Science in Population Health City University of New York New York, NY United States; 2 Division of General Internal Medicine Department of Medicine Montefiore Health System, Albert Einstein College of Medicine Bronx, NY United States; 3 Department of Statistics and Biostatistics Rutgers University New Brunswick, NJ United States; 4 Department of Psychology University of Massachusetts Boston, MA United States; 5 The Fenway Institute Fenway Health Boston, MA United States; 6 Center for Alcohol and Addiction Studies Brown University Providence, RI United States; 7 Hunter HIV/AIDS Research Team Department of Psychology, Hunter College City University of New York New York, NY United States; 8 The Foundation for AIDS Research New York, NY United States

**Keywords:** HIV, pre-exposure prophylaxis, implementation science

## Abstract

**Background:**

Gay, bisexual, and other men who have sex with men continue to bear a large burden of the HIV epidemic in the United States and are among the only populations with increasing incidence in recent years.

**Objective:**

The *Together 5000* (T5K) Study aimed to enroll a US-based, racially diverse sample of HIV-negative men, transmen, and transwomen who are not on pre-exposure prophylaxis (PrEP) into an observational cohort to inform the design, implementation, scale-up, and evaluation of HIV prevention programs.

**Methods:**

We used internet-based strategies to enroll a large, racially diverse national sample of HIV-negative men, transmen, and transwomen aged 16 to 49 years at high risk of HIV acquisition via sexual networking apps. Study participants are contacted every 6 months (in between annual surveys) for a brief survey on HIV testing, HIV diagnosis, and PrEP use (ie, attempts to access, PrEP initiation, and PrEP discontinuation). Participants complete annual self-administered at-home HIV testing and Web-based surveys. Using baseline serologic data and self-reported HIV testing history, we reconstructed a cohort of persons who were HIV negative at 12 months before baseline to estimate HIV incidence leading up to cohort enrollment.

**Results:**

The study sample included 8777 participants from all 50 US states, Puerto Rico, and Guam; 50.91% (4468/8777) were persons of color and 25.30% (2221/8777) were young individuals aged 16 to 24 years. Per eligibility criteria, all T5K participants reported having sex with >2 male partners in the 90 days before enrollment, self-reported not having been diagnosed with HIV, and were not actively taking PrEP. In addition, 79.39% (6968/8777) reported >2 insertive condomless anal sex (CAS) acts, 61.02% (5356/8777) reported >1 receptive CAS acts in the past 90 days. Furthermore, most (7525/8777, 85.74%) reported never having taken PrEP. In total, 70.25% (6166/8777) were sent a self-administered at-home HIV test kit and 82.29% (5074/6166) of those sent a kit returned a sample for testing. The HIV incidence rate during the 12-month period leading up to enrollment was estimated to be 2.41 (95% CI 2.02-2.90) per 100 person-years.

**Conclusions:**

A large, national, and racially diverse fully Web-based cohort of HIV-negative men, transmen, and transwomen at high risk for HIV seroconversion has successfully been recruited into longitudinal follow-up. This cohort is at high risk for HIV acquisition and can provide important insights related to the real-world uptake, impact, and equity of HIV prevention interventions in the United States. Participants can be invited to participate in trials aimed at testing strategies to improve the uptake of and engagement in these interventions.

**International Registered Report Identifier (IRRID):**

RR1-10.2196/13715

## Introduction

Gay, bisexual, and other men who have sex with men (GBM) continue to bear the burden of the HIV epidemic in the United States and are among the only populations with increasing incidence in recent years [1]. The high rate of HIV incidence among GBM in the United States and the unabated racial and ethnic disparities in the era of HIV pre-exposure prophylaxis (PrEP) highlight the urgent need to understand more about PrEP uptake and missed prevention opportunities and their drivers [2]. Presently, PrEP is recommended by the Centers for Disease Control and Prevention [3,4] to prevent new HIV infections and has been supported by local health departments in many major US cities [5,6]. However, optimal implementation and delivery strategies that provide greater access to those in need of biomedical HIV prevention interventions have not been identified and may vary substantially by population, setting, and policy environment. Recent data suggest that, despite accounting for nearly half of all US HIV infections, black men represent fewer than 10% of those taking PrEP. In contrast, white men made up 27% of those infected with HIV in 2014 but accounted for 75% of those taking PrEP in 2015 [7].

Most data on PrEP uptake are based on insurance claims or pharmacy prescriptions for emtricitabine and tenofovir disoproxil fumarate. A major limitation of available insurance plan–based or even population-based data on PrEP uptake [8] is that they do not provide epidemiological or behavioral information on the underlying population of persons in *need* of PrEP. Thus, there is limited ability to assess both PrEP coverage and the major barriers and facilitators of PrEP uptake among those at the highest risk for HIV acquisition. Importantly, many individuals who are most in need of PrEP may not have regular encounters with or access to health care and thus may not be reachable via health care providers or other conventional provider-based intervention targeting strategies. Specifically, the most common way that US GBM meet sexual partners is via the internet, with a rapid and recent shift to the use of geosocial sexual networking mobile apps, making these platforms particularly important both for understanding barriers to PrEP uptake and targeting interventions [9-11]. We describe the protocol and baseline participant characteristics for the *Together 5000* (T5K) cohort study.

In response to a 2016 request for applications from the US National Institutes of Health (NIH) [12], we sought to recruit, via sexual networking apps, a racially and geographically diverse sample of HIV-negative men, transmen, and transwomen who have sex with men who are not on PrEP to better inform the design, implementation, scale-up, and evaluation of HIV prevention programs.

## Methods

### Target Population

The T5K cohort study used established ([13]; also CG et al, unpublished data, 2019) internet-based strategies to enroll a large sample of HIV-negative men, transmen, and transwomen who have sex with men aged 16 to 49 years and are at high risk of HIV acquisition. The cohort will be followed prospectively for 48 months for the outcomes of PrEP uptake and HIV seroconversion. We aimed to enroll a cohort of participants at high risk for HIV that was geographically diverse (ie, representing every US state and territory), racially and ethnically diverse (4468/8777, 50.91% participants of color), and young (2221/8777, 25.30% aged 16-24 years). We achieved these goals without needing to employ stratified sampling.

### Cohort Eligibility and Recruitment

**Figure 1:**
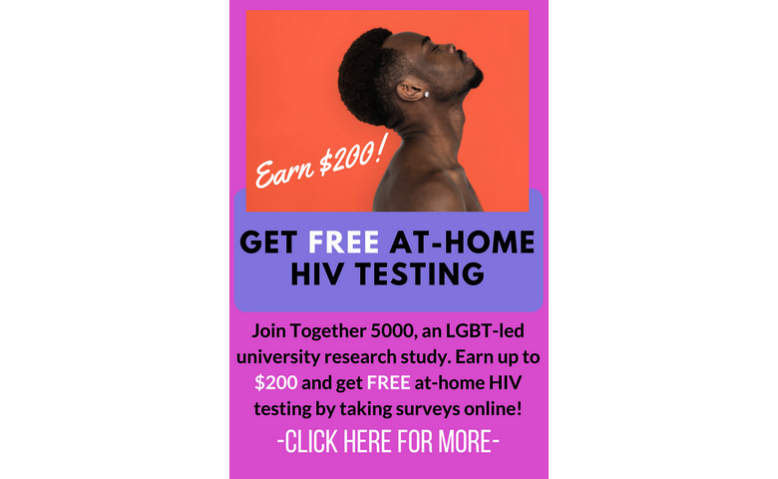
*Together 5000* example recruitment advertisement.

Open enrollment for T5K began in October 2017 and concluded in June 2018, when 67,166 of the estimated 649,000 (67,166/649,000, 10.35%) males eligible for PrEP across the United States were using it [8]. Participants were recruited via ads on men-for-men geosocial sexual networking mobile phone apps (Figure 1). Although not the targeted audience, transgender women and men were not excluded if they reported sex with men and otherwise met the eligibility criteria (Table 1). The study was promoted as an opportunity to receive at-home, self-administered HIV testing. Advertisements were geotargeted to individuals using apps inside the United States and the US territories.

**Table 1:** Eligibility criteria for the *Together 5000* cohort study.

Eligibility criteria		Participants (N=8777), n (%)
**Core eligibility criteria (all participants must meet all of these criteria)**		
	Aged 16 to 49 years	8777 (100.00)
	At least 2 male sex partners in the past 90 days	8777 (100.00)
	Not currently participating in a clinical trial for an HIV vaccine or pre-exposure prophylaxis	8777 (100.00)
	Not currently on pre-exposure prophylaxis	8777 (100.00)
	Never diagnosed with HIV (self-report)	8777 (100.00)
	Currently residing in the United States or territories	8777 (100.00)
	Not cisgender female	8777 (100.00)
**Additional eligibility criteria (participants must meet at least 1)**		
	>1 receptive condomless anal sex acts with a male partner in the last 3 months	6968 (79.39)
	>2 insertive condomless anal sex acts with a male partner in the last 3 months	5356 (61.02)
	Used methamphetamines in the last 3 months	1058 (12.05)
	Rectal gonorrhea/chlamydia in the last 12 months	684 (7.79)
	Syphilis diagnosis in the last 12 months	402 (4.58)
	Used postexposure prophylaxis in the last 12 months	219 (2.50)
	Shared injection drug needles in the last 12 months	180 (2.05)

Potential participants were directed to a secure enrollment survey in their device’s Web browser and presented with a screen describing study participation and eliciting informed consent. The informed consent described the incentive schedule: US $15 for completing a secondary survey (ie, 1 after the enrollment survey) if they were eligible and another US $15 for completing self-administered at-home HIV testing (ie, oral fluid sample returned to the study laboratory for testing). Additional incentives, described in the informed consent, are available to participants who complete prospective longitudinal follow-up assessments.

### Enrollment

Interested individuals were screened for eligibility via a Web-based survey collecting data on sexual behavior, substance use, demographic characteristics, history of PrEP use, and history of postexposure prophylaxis (PEP) use. The survey was programmed into Qualtrics survey software and tested by study staff. Measures had been previously used by the members of the research team or were derived from the published research. Our Web-based survey was divided into thematic blocks based on question content and used adaptive questions based on survey responses from the participants. Examples include survey questions about known HIV status, PrEP use, and main sexual partners. Survey items were not randomly ordered by participant. The number of items answered by the participants and displayed per page of the Web-based survey varied by subject and participant responses because of the skip and/or display logic used within the survey. However, to improve ease of use on mobile devices and reduce survey fatigue, each page contained 1 to 2 questions. Participants could not click back to view or change a previous response because of the skip and/or display logic depending on previous responses. If a participant chose an incorrect response, they could contact the study staff to reset that response. Before activating the survey, members of the study staff tested the survey extensively for usability and technical function. The survey was tested on Windows, Mac, iOS, and Android devices and on Chrome, Firefox, Internet Explorer, Opera, and Safari Web browsers. Eligible and consenting individuals were asked to provide contact information for longitudinal follow-up. All participants were assigned a unique identifier at study enrollment and this unique identifier was used for all study databases and datasets. Participants’ contact information was stored in an encrypted database separated from their questionnaire answers and other study-related information. Only designated study staff were allowed access to study databases.

Enrolled individuals were sent a secondary Web-based survey that assessed the psychosocial characteristics. As the study participation involved receiving and returning an at-home HIV test kit via mail, as well as follow-up HIV test kits, consenting participants were required to provide name, mailing address, email address, and other contact information. We followed established and effective measures to minimize repeat participation and fraudulent manipulation of HIV testing procedures, including recording internet protocol addresses of participants and using cookies to block repeated attempts (CG et al, unpublished data, 2019). Our enrollment survey blocked multiple submissions, our databases flagged duplicate contact information, and all mailing addresses were validated with the US postal service. Multiple entries were identified by email addresses and/or phone numbers. In addition, the data manager manually checked for duplicate entries during baseline data collection. We also assessed time to completion of our Web-based surveys and checked for variability in response sets.

Upon completing this secondary survey, participants were mailed an OraSure HIV-1 specimen collection device to use at home. Participants were also provided access to a study video along with printed instructions on completing the HIV test, as well as our phone number in case they had questions. Procedures involved taking an oral swab and placing it in an oral fluid container and mailing the specimen using provided prepaid shipping materials to the Wadsworth Center Laboratory of the New York State Department of Health for antibody testing (Avioq HIV-1 Microelisa System) and archiving. Participants indicated the date of collection and any samples received by the lab after 21 days were not analyzed. In these instances, participants were contacted to retest. Median number of days between specimen collection and lab receipt was 4 days (interquartile range 3 to 6 days).

### Participants With Unknown Baseline HIV Status

Participants who enrolled and completed a baseline questionnaire but did not return an HIV test kit (baseline serostatus unknown) will continue to be followed and asked at regular intervals to submit an oral fluid sample for HIV testing using the at-home sampling kit. For the purposes of prospectively estimating HIV incidence in the T5K cohort, these individuals will be excluded. However, we will conduct post hoc sensitivity analyses that make assumptions about having similar, lower, or higher HIV risk profiles than the T5K participants for whom baseline HIV status was determined.

### HIV Incidence in the 12 Months Before Cohort Enrollment

We estimated pre-enrollment HIV incidence using baseline data on HIV serostatus and HIV testing history from T5K participants. Using these data, we reconstructed a cohort of individuals who could all be classified as HIV negative as of 12 months before cohort enrollment. For those HIV positive at enrollment, we estimated the time of seroconversion using self-reported data on the timing of the last HIV-negative test. Specifically, those self-reporting a negative HIV test within 6 months of T5K enrollment were classified as being HIV negative as of 6 months before enrollment, with seroconversion timing assumed to be distributed evenly during the 6 months leading up to study enrollment. Similarly, participants self-reporting a negative HIV test 7 to 12 months before T5K enrollment were classified as being HIV negative as of 12 months before enrollment, with seroconversion timing assumed to be distributed evenly during the 12 months leading up to study enrollment. We also estimated HIV incidence for the 6-month period leading up to cohort enrollment. For the purposes of identifying boundaries around minimum and maximum incidence rates, we conducted sensitivity analyses representing assumptions of the timing of seroconversion at the extremes.

### Sample Size and Statistical Power

Enrollment in T5K had a targeted sample size of 5000 participants with a confirmed HIV-negative test for prospective follow-up. The 5000 number was chosen to provide precise estimates of HIV incidence of 1.11 per 100 person-years (193 seroconversions of 17,329 person-years of follow-up over 48 months). This sample size also allows 80% power to detect binary exposures with frequencies between 20% and 80% for adjusted relative hazards ranging from 1.73 to 2.27 as statistically significant.

### Data Management and Analysis

All data from the Web-based surveys and the laboratory testing were imported, cleaned, and merged using SAS. Data were geocoded to an exact address or Zone Improvement Plan (ZIP) code. Maps were created in ArcGIS and did not include exact participant location.

### Ethical Approval

The T5K study protocol was approved by the Institutional Review Board of the City University of New York (CUNY) Graduate School of Public Health and Health Policy.

## Results

### Cohort Eligibility and Recruitment

**Figure 2:**
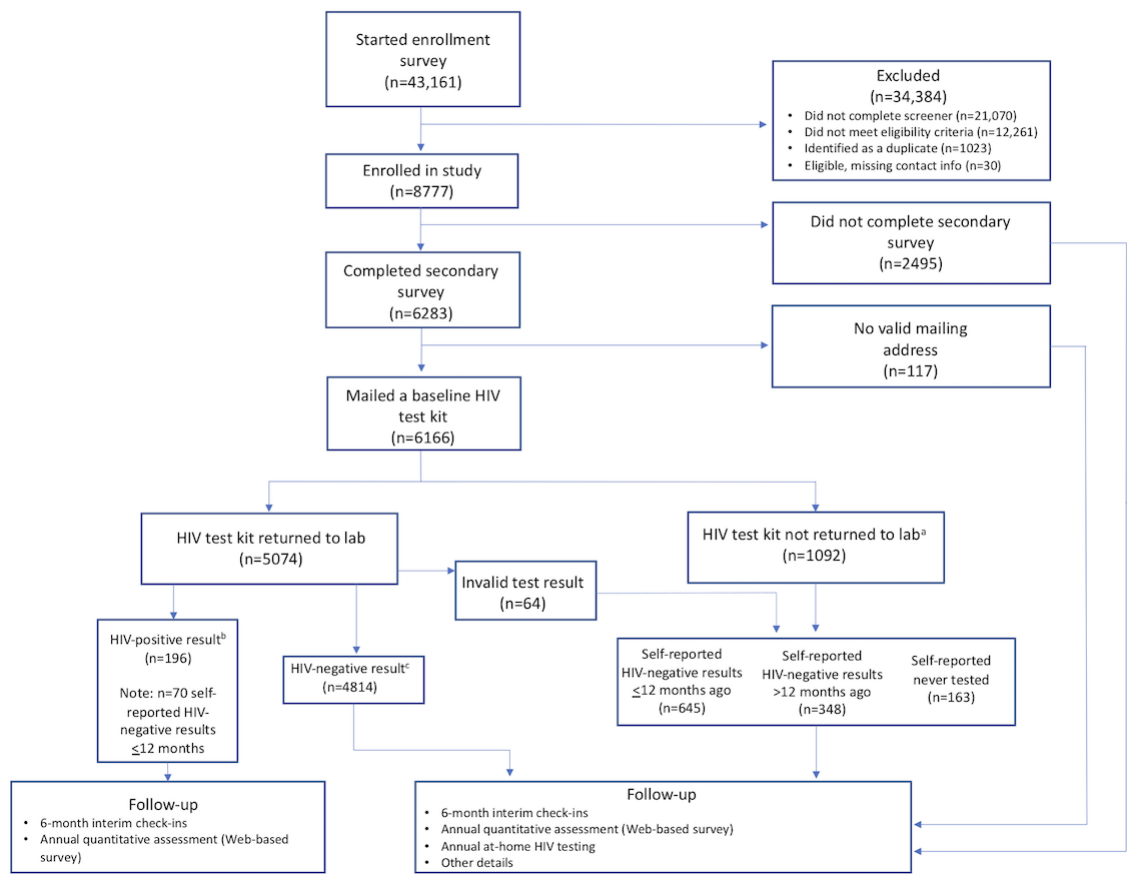
Consolidated Standards Of Reporting Trials diagram illustrating enrollment in the *Together 5000* cohort study. (a) 9 participants told us they tested HIV-positive outside of the study while we were in the process of trying to collect an HIV test kit from them. These participants declined to complete testing with us. (b) One participant who tested HIV-positive with our test reported an HIV-negative result from outside of the study. (c) One participant who tested HIV-negative with our test reported an HIV-positive test from outside of the study.

In total, 43,161 individuals began our enrollment survey and 22,091 (22,091/43,161, 51.18%) completed it (Figure 2). Of the noncompleters (N=21,070), 61.04% (12,862/21,070) closed their browser window on the informed consent page (ie, immediately). Of the completers (N=22,091), 9193 (9193/22,091, 41.61%) were eligible; however, 1023 were excluded because we determined their response to be a duplicate entry. Of the remaining 8807 participants who provided informed consent to participate in the study, 30 were excluded, as they did not provide contact information.

### Enrollment

**Figure 3:**
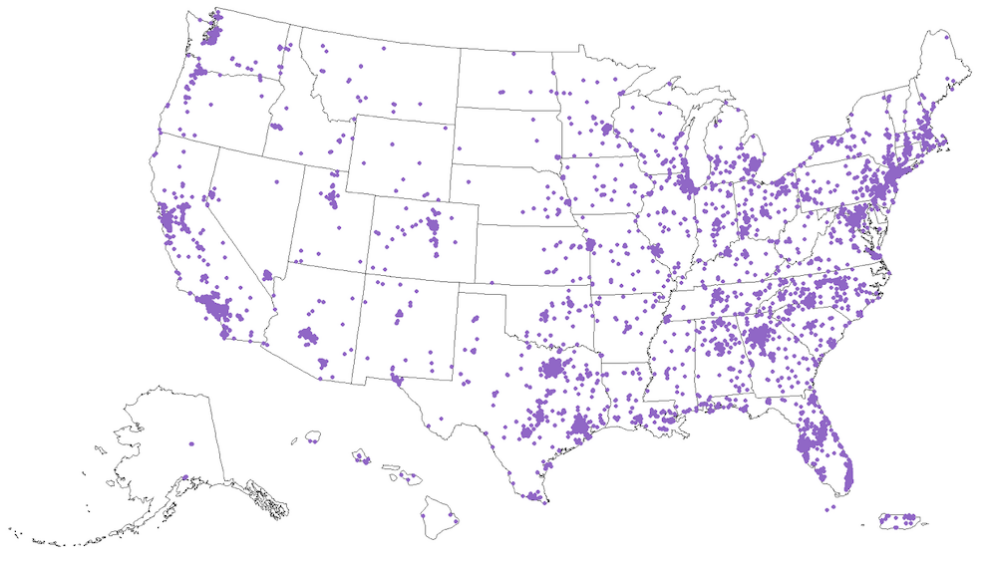
Geographic distribution of the *Together 5000* cohort.

The final sample was 8777 consented participants from all 50 US states, Puerto Rico, and Guam (Figure 3). The descriptive statistics of the cohort and HIV incidence rates calculated included responses from enrolled participants meeting the eligibility criteria and completing questionnaires.

### Baseline Characteristics of the *Together 5000* Cohort

The final cohort of 8777 individuals was geographically diverse, including participants from all 50 US states, Puerto Rico, and Guam (Figure 3). Nearly all (8554/8777, 97.46%) were cisgender male, 0.72% (63/8777) were transgender women, and 0.60% (53/8777) were transgender men who have sex with men (Table 2). There were also 107 (107/8777, 1.22%) individuals who self-identified outside of the gender binary—all reported being assigned male sex at birth. In total, 50.91% (4468/8777) were persons of color, and 25.30% (2221/8777) were young individuals aged 16 to 24 years.

**Table 2:** Characteristics *Together 5000* participants at the time of enrollment.

Variable		Participants (N=8777), n (%)	Never on PrEP^a^ (N=7525), n (%)	History of PrEP (N=1252), n (%)	Chi-square value (*df*)	*P* value
**Race/ethnicity**					10.2 (4)	.04
	White	4309 (49.09)	3672 (48.80)	637 (50.88)	—^b^	—
	Black	1156 (13.17)	1009 (13.41)	147 (11.74)	—	—
	Latino	2227 (25.37)	1932 (25.67)	295 (23.56)	—	—
	Asian/Pacific Islander	311 (3.43)	253 (3.36)	58 (4.63)	—	—
	Multiracial/other	774 (8.82)	659 (8.76)	115 (9.19)	—	—
**Gender**					16.0 (3)	.001
	Cis male	8554 (97.46)	7343 (97.58)	1211 (96.73)	—	—
	Transwoman	63 (0.72)	59 (0.78)	4 (0.32)	—	—
	Transman	53 (0.60)	44 (0.58)	9 (0.72)	—	—
	Nonbinary (male at birth)	107 (1.22)	79 (1.05)	28 (2.24)	—	—
**Sexual identity**					39.6 (2)	<.001
	Gay identified	7314 (83.33)	6194 (82.31)	1120 (89.46)	—	—
	Bisexual identified	1346 (15.33)	1223 (16.25)	123 (9.82)	—	—
	Neither/other	117 (1.33)	108 (1.44)	9 (0.72)	—	—
**Age (years)**					98.2 (6)	<.001
	16-19	517 (5.89)	498 (6.62)	19(1.52)	—	—
	20-24	1704 (19.41)	1523 (20.24)	181 (14.46)	—	—
	25-29	2360 (26.89)	2003 (26.62)	357 (28.51)	—	—
	30-34	1664 (18.95)	1373 (18.25)	291 (23.24)	—	—
	35-39	1186 (13.51)	971 (12.90)	215 (17.17)	—	—
	40-44	738 (8.41)	625 (8.31)	113 (9.03)	—	—
	45 and older	608 (6.93)	532 (7.06)	76 (6.07)	—	—
No health insurance^c^ (valid N=6283)		1575 (25.07)	1363 (25.32)	212 (23.56)	2.9 (1)	.23
**HIV risk factors**						
	Recent sexually transmitted infection diagnosis (last 12 months)	1629 (18.56)	1243 (16.52)	386 (30.83)	145.5 (1)	<.001
	Syphilis in lifetime	1359 (15.48)	1042 (13.85)	317 (25.32)	108.0 (1)	<.001
	Syphilis in the last 12 months	402 (4.58)	303 (4.03)	99(7.91)	115.8 (1)	<.001
	Rectal gonorrhea/chlamydia in the last 12 months	684 (7.79)	489 (6.50)	195 (15.58)	332.5 (1)	<.001
	Oral gonorrhea/chlamydia in the last 12 months	1309 (14.91)	958 (12.73)	351 (28.04)	198.1 (1)	<.001
	>1 receptive condomless anal sex acts with male in the last 3 months	6968 (79.39)	5973 (79.38)	995 (79.47)	0.01 (1)	.94
	>2 insertive condomless anal sex acts with male in the last 3 months	5356 (61.02)	4545 (60.40)	811 (64.78)	8.6 (1)	.003
	Having taken PEP^d^ in the last 12 months	219 (2.50)	93 (1.24)	126 (10.06)	733.5 (1)	<.001
	Methamphetamine use in the last 3 months	1058 (12.05)	875 (11.63)	183 (14.62)	9.0 (1)	.003
	Sharing needles in the last 12 months	180 (2.05)	144 (1.91)	36 (2.88)	6.1 (1)	.05
	Sex work in the last 3 months	1339 (15.26)	1127 (14.98)	212 (16.93)	3.2 (1)	.08
No primary health care provider^c^ (valid N=6283)		3057 (48.66)	2700 (50.15)	357 (39.67)	34.0 (1)	<.001
Disclosed sexual behavior to health care provider^e^ (valid N=3226)		2343 (72.63)	1848 (78.87)	495 (91.16)	112.1 (1)	<.001
Never heard of PrEP		476 (5.42)	476 (6.33)	0 (0.0)	—	—
**HIV** **testing history**					414.9 (2)	<.001
	<12 months ago	5362 (61.09)	4277 (56.84)	1085 (86.67)	—	—
	12 or more months ago	2292 (26.11)	2145 (28.50)	147 (11.74)	—	—
	Never had an HIV test	1123 (12.79)	1103 (14.66)	20 (1.60)	—	—

^a^PrEP: pre-exposure prophylaxis.

^b^Not applicable.

^c^Among those who completed our secondary survey.

^d^PEP: postexposure prophylaxis.

^e^Among those who completed our secondary survey and reported having a health care provider.

#### HIV Risk

Per eligibility criteria, at enrollment, all T5K participants reported sex with >2 male partners in the 90 days before enrollment, were HIV negative, and were not taking PrEP. In addition, 79.40% (6969/8777) reported >2 insertive condomless anal sex (CAS) acts, 61.02% (5356/8777) reported >1 receptive CAS acts in the past 90 days (Table 2), 2.50% (219/8777) reported having taken HIV PEP in the last 12 months, 18.56% (1629/8777) reported having a sexually transmitted infection (STI) diagnosis in the last 12 months, including rectal (684/8777, 7.79%) or oral (1309/8777, 14.91%) gonorrhea/chlamydia, and 15.48% (1359/8777) reported a lifetime syphilis diagnosis. However, only 14.26% (1252/8777) reported ever having taken PrEP. Nearly half (3057/6283, 48.66%) reported not having a primary health care provider, and 12.79% (1123/8777) said that they had never tested for HIV.

#### History of Pre-Exposure Prophylaxis Use

In Table 2, we compare participants who reported never having taken PrEP (7525/8777, 85.74% of enrolled) with those who reported having taken PrEP previously (1252/8777, 14.26% of enrolled). Previous PrEP users were significantly more likely to be white, gay-identified, and older and have tested for HIV in the last 12 months—as well as reported significantly more HIV risk factors (past year STI diagnosis, past year PEP use, and >2 insertive CAS acts with male partners in the last 3 months). Participants with a history of PrEP use were significantly more likely to have used PEP (10.1% vs 1.2%; *P*<.001) and have had a primary health care provider (and be open about their sexual behavior with men to that provider).

#### Completion of Baseline HIV Test

Of the 8777 participants enrolled, 6166 (70.25%) provided valid mailing addresses on the second survey and were sent an at-home HIV testing kit, and 5074 participants provided a sample to test (5074/6166, 82.29% of those sent a kit and 5074/8777, 57.81% of those enrolled). Compared with participants who did not provide a sample for testing (3703), those who did provide a sample (5074) were significantly more likely to report income >US $50,000 (22.12% vs 26.25%; *P*<.001), be white (43.64% vs 53.07 *P*<.001), have a college degree (29.78% vs 40.69%; *P*<.001), and be slightly older on average (29.81 vs 30.84 years; *P*<.001).

#### HIV Status and Estimating the Cohort HIV Incidence Rate Before *Together 5000* Study Enrollment

Table 3 describes the outcomes of baseline HIV testing. Of 5074 persons who returned their test kit, 196 (196/5074, 3.86%) had undiagnosed HIV. Of those 196, individuals self-reported that (1) they had a negative HIV test within 6 months of T5K enrollment (n=34), (2) they had a negative HIV test within 7 to 12 months of T5K enrollment (n=36), (3) they had a negative HIV test more than 12 months before T5K enrollment (n=95), or (4) they had never tested for HIV before T5K enrollment (n=31). As an upper bound, we calculated the incidence rate for the extreme scenario that assumes all 196 persons seroconverted during the 12 months before study enrollment (Table 4). Using these approaches, we estimated that the incidence rate in this cohort in the 12-month period leading up to T5K study enrollment was 2.42 (95% CI 2.02-2.90) per 100 person-years. The estimate for the 6-month period leading up to enrollment was 2.16 (95% CI 1.63-2.81). Alternate scenarios under different assumptions about the timing of seroconversion for persons with either less recent HIV tests or no history of HIV testing gave slightly higher incidence estimates, ranging from 2.74 to 3.76 per 100 person-years. The maximum HIV incidence estimate was 3.98 (95% CI 3.45-4.57) per 100 person-years, which assumed that all 196 persons testing HIV positive had seroconverted in the 12 months before study enrollment (Multimedia Appendix 1). Although no participants were on PrEP at enrollment, when comparing the crude prestudy incidence rates for those never on PrEP to those with a history of PrEP, the incidence rate was higher for both the 6-month (incidence rate ratio [IRR]: 1.62; 95% CI 0.69-4.61) and the 12-month (IRR: 2.35; 95% CI 1.21, 5.20) periods before enrollment.

**Table 3:** HIV testing outcomes among *Together 5000* study population.

Variable				Total participants (N=8777), n (%)	Never on PrEP^a^ (N=7525), n (%)	History of PrEP N=1252), n (%)	Chi-square value *(df)*	*P* value
**Returned HIV test kit to the lab**				5074 (57.81)	4343 (57.71)	731 (58.39)	0.2 (1)	.66
	**HIV test-kit results^b^**			—^e^	—	—	14.6 (1)	<.001
		HIV-negative test result		4814 (94.88)	4101 (94.43)	713 (97.54)	—	—
		HIV-positive test result		196 (3.86)	186 (4.28)	10 (1.37)	—	—
		**Date of most recent HIV test^d^**		—	—	—	5.0 (3)	.18^e^
			Self-reported HIV-negative test <6 months ago	34 (17.35)	30 (16.12)	4 (40.00)	—	—
			Self-reported HIV-negative test 7-12 months ago	36 (18.37)	34 (18.28)	2 (20.00)	—	—
			Self-reported HIV-negative test >12 months ago	95 (48.47)	91 (48.92)	4 (40.00)	—	—
			No previous HIV test	31 (15.82)	31 (16.67)	0 (0.00)	—	—

^a^PrEP: pre-exposure prophylaxis.

^b^N=n value from returned HIV test.

^c^Not Applicable.

^d^N=n value from HIV-positive test result.

^e^Mid *P* exact test.

**Table 4:** HIV incidence estimates among *Together 5000* study population in the 12- and 6-month periods before study enrollment.

Period before enrollment		All participants	No history of PrEP^a^ use	History of PrEP use	Incidence rate ratio
**12-month period before enrollment**					
	Number of presumed recent seroconversions, n (%)	118 (100)	110 (93)	8 (7)	—^b^
	Person-years at risk among seroconverters	58.75	54.75	4	—
	Person-years at risk among HIV-negative persons	4814	4101	713	—
	Incidence rate per 100 person years (95% CI)	2.41 (2.02-2.90)	2.63 (2.19-3.19)	1.12 (0.52-2.13)	2.36 (1.21-5.20)
**6-month period before enrollment**					
	Number of presumed recent seroconversions, n (%)	52 (100)	47 (90)	5 (10)	—
	Person-years at risk among seroconverters	13.0	11.8	1.3	—
	Person-years at risk among HIV-negative persons	2407	2051	357	—
	Incidence rate per 100 person-years (95% CI)	2.15 (1.63-2.81)	2.28 (1.70-3.01)	1.40 (0.52-3.12)	1.63 (0.69-4.61)

^a^PrEP: pre-exposure prophylaxis.

^b^Not applicable.

### Longitudinal Follow-Up and Measurements

Prospective closed follow-up of T5K participants includes completion of an annual self-administered at-home HIV testing and extensive Web-based surveys beginning 12 months after the baseline survey. In addition, participants will be contacted every 6 months (in between annual surveys) for a brief survey on HIV testing, diagnosis, and PrEP use (ie, attempts to access PrEP, PrEP initiation, PrEP adherence, and PrEP discontinuation). Participants who self-report being on PrEP are asked to provide proof in the form of a picture of the medication bottle with their prescription information. A comprehensive list of key study measurements by study wave is included in Table 5. We will follow the cohort of 8777 individuals for up to 4 years to characterize the following: the rate of PrEP uptake/discontinuation; individual /network /contextual-level determinants of PrEP uptake /discontinuation; patterns of PrEP use (eg, daily or on demand); the rate of new HIV seroconversions and other missed HIV prevention opportunities (ie, STIs while not on PrEP); individual/network/contextual-level determinants of HIV seroconversion and missed HIV prevention opportunities; racial/ethnic disparities in HIV incidence and their trends over time; and the influence of PrEP uptake on racial/ethnic disparities in HIV incidence.

**Table 5:** Follow-up measures in the *Together 5000* cohort.

Measures^a^	Number of items	Administered at					
		Enrollment survey	Secondary survey	12 months	24 months	36 months	48 months
Sociodemographic questionnaire	14	X^b^	—^c^	X	X	X	X
PrEP^d^ and PEP^e^ history	5	X	—	X	X	X	X
Men who have sex with men risk index^f^	10	X	—	X	X	X	X
History of sexually transmitted infections and HIV testing	13	X	—	X	X	X	X
Main sexual partner	7	X	—	X	X	X	X
Drug, alcohol, and cigarette use	4	X	—	X	X	X	X
Incarceration and recent arrest	3	—	X	X	X	X	X
Connor-Davidson resilience scale	10	—	X	X	X	X	X
Alcohol, smoking, and substance involvement screening test	13-86	—	X	X	X	X	X
Alcohol use disorder identification test	10	—	X	X	X	X	X
Generalized anxiety disorder	2	—	X	X	X	X	X
Patient health questionnaire	2	—	X	X	X	X	X
Internalized homophobia scale	14	—	X	X	X	X	X
Lesbian, gay, bisexual, and transgender resources and policies	14	—	X	X	X	X	X
General PrEP experiences and acceptability	12	—	X	X	X	X	X
Barrier to PrEP uptake	14	—	X	X	X	X	X
Partner violence questionnaire	12	—	X	X	X	X	X
Hepatitis C virus risk score	6	—	X	X	X	X	X
Multidimensional scale of perceived social support	12	—	X	X	X	X	X
Multidimensional peer-victimization scale^g^	14	—	X	—	—	—	—
Position preference	1	—	X	—	—	—	—
Sexual debut and childhood sexual abuse	7	—	X	—	—	—	—

^a^There are brief check-in surveys at 6, 18, 30, and 42 months.

^b^Construct is assessed.

^c^Construct is not assessed.

^d^PrEP: pre-exposure prophylaxis.

^e^PEP: postexposure prophylaxis.

^f^Modified to include questions about female, transmale, and transfemale sex partners and condomless sex acts with female sex partners.

^g^Provided as published scale to currently enrolled high school students, modified from *past year* to *when you were in high school* for older participants, added 2 questions regarding missing school in the last year and whether the scale items occurred on the Web.

Given the novel nature of this entirely Web-based nationwide cohort, we elected to longitudinally follow *all* participants providing consent at enrollment (N=8777), rather than only those who completed HIV testing. Participants who did not complete the secondary survey or subsequent HIV testing will have the opportunity to provide those data/samples at future assessments, and this will enable us to learn more about differential rates of participation and attrition in the cohort moving forward.

#### Observed Seroconverters

Participants who were HIV negative at baseline and who indicate that they tested HIV positive between study assessments will be asked and incentivized to provide HIV status documentation and will not be asked to complete additional HIV tests for study purposes. These participants will be classified as recent seroconverters. Among the remaining participants, those who test positive in the 12-month home test will also be classified as recent seroconverters, and those who test negative will be classified as remaining seronegative. We will contact recent seroconverters to capture information about and facilitate the process of linkage to care. In addition to referral for treatment, approximately 3 months after their diagnosis via the study, we will invite a sample of seroconverting participants to complete a semistructured individual telephone interview to identify missed HIV prevention opportunities and barriers/facilitators of their entry into HIV care and subsequent retention. We will follow these participants until the end of the study to document movement through the HIV care continuum [14,15].

## Discussion

### Principal Objectives

We have successfully recruited an entirely Web-based national cohort of confirmed HIV-negative men, transgender men, and transgender women who have sex with men and are at very high risk for HIV into longitudinal follow-up. Importantly, none of the T5K cohort members were on PrEP at enrollment but all met the objective criteria for PrEP use. This will allow our study to fill critical knowledge gaps that can improve the understanding of barriers to PrEP uptake and engagement among those most in need of HIV prevention interventions. This cohort also offers opportunities to examine the effect of different implementation strategies aimed at improving the uptake of and engagement with HIV prevention interventions, including those that can be delivered on the Web.

We observed a higher rate of several recent and lifetime STIs among persons with a history of PrEP compared with those who were never on PrEP. This could represent differences in the risk profile of persons who have used PrEP (ie, they were at higher risk for an STI in the past before or while using PrEP). However, many STIs are asymptomatic, making it likely that the STIs reported by this group at baseline would have been diagnosed as a result of them having initiated PrEP in the past, as PrEP services involve baseline and follow-up STI screening and treatment. We also observed that those with a history of PrEP were >8 times more likely to have taken PEP in the past. This could reflect better access to HIV prevention services or greater HIV risk among those with a history of PrEP use. Persons on PEP are usually good candidates for PrEP, and PrEP should be systematically discussed and offered to all persons completing PEP if there is a likelihood of ongoing HIV risk.

Our estimates of HIV incidence in the 12-month period leading up to T5K cohort enrollment (2.4% per year overall and 2.6% per year among those never on PrEP) suggest that the risk of HIV acquisition that we will observe prospectively will also be quite high, unless PrEP uptake increases dramatically. Indeed, we observed that HIV incidence was substantially higher among those never on PrEP compared with those with a history of PrEP before enrollment (IRR 2.36; 95% CI 1.21-5.20).

### Strengths

Major strengths of this cohort study include the large sample size, the wealth of self-reported information related to HIV risk and PrEP, direct measurement of HIV status and seroconversion, geographic representativeness, recruitment of participants independent of their access to/engagement with the health care system, and inclusion of large numbers of racial/ethnic minority GBM as well as those aged <25 years. Having every US state, Puerto Rico, and Guam represented allows for a robust exploration of state-level policies and other higher-level effects (ie, contextual factors) as potential determinants of PrEP uptake and HIV risk. More than 50% (4468/8777, 50.91%) of the cohort comprises HIV-negative men of color, allowing in-depth investigations into the mechanisms of racial/ethnic disparities in PrEP uptake, HIV incidence, and circumstances surrounding HIV seroconversion. Importantly, the T5K cohort study design, with semiannual Web-based at-home surveys and at-home self-sampling for HIV testing, reduces the potential for participation and questionnaire-response bias introduced by the Hawthorne effect, which could be stronger in face-to-face studies. Studies involving frequent face-to-face contact can cause participants to adopt behaviors that make them less representative of the high-risk populations from which they were drawn. Of note, McCabridge et al [16] introduced the construct of *research participant effects* that was built upon the Hawthorne effect [17] by elaborating on the implications of research on the mechanisms that introduce bias, including demand characteristics [18,19]. Studies involving high levels of staff contact with participants may induce behavior change by repeatedly engaging participants outside of their natural context, artificially influencing results [17,19,20] and reducing generalizability [21].

### Weaknesses

Recruiting participants and gathering data on the Web is a much less controlled research environment than face-to-face or telephone interview studies. Web-based recruitment also increases our vulnerability to repeat participation and fraudulent manipulation of HIV testing procedures (eg, someone else’s saliva, other than that of the enrolled participant, could be submitted to the lab). However, we followed established and effective measures to minimize these risks [22,23]. Although we took steps to assess whether participants were reading the interview questions, participants recruited on the Web may be less cognitively engaged in questionnaire completion and less inclined to provide a specimen than if they were interacting directly with study personnel. Our study population was recruited via sexual networking apps and represented a sample of those at high risk for HIV, who are not on PrEP at cohort enrollment. However, the underlying population that gave rise to the T5K cohort is not representative of all HIV-negative GBM at high risk for HIV in the United States [24]. Finally, HIV seroconversions and PrEP uptake may be incompletely ascertained, and the timing of HIV seroconversions must be estimated using a midpoint approach with broad intervals, potentially introducing bias in our baseline HIV incidence estimates. For example, our study eligibility/inclusion criterion of *never diagnosed with HIV (self-report)* effectively excludes frequent HIV testers who seroconverted and were diagnosed in the period immediately before study launch.

### Challenges and Lessons Learned

There have been many challenges and lessons learned in the launch and execution of the T5K study, which have been detailed elsewhere ([13]; also CG et al, unpublished data, 2019). In brief, although there are many advantages to entirely Web-based studies, challenges we have encountered include greater difficulty obtaining signed informed consent, returning HIV test results to participants, linking persons to HIV services when needed (especially in underserved areas), the potential for participants to be distracted, and difficulty ensuring unique and valid participants—and new challenges with regard to privacy and data security.

### Applications for Clinical and Population Health Intervention Studies

Intervention studies are of great interest to the T5K study team. Our goal is to utilize key findings from this cohort to develop interventions, potentially including some delivered completely on the Web, once the observational phase is completed. There will likely be opportunity for natural experiment design studies that allow for rigorous examination of future state or national policy changes, an introduction of long-acting PrEP formulations or dosing recommendations, and other novel HIV prevention modalities. To facilitate such research, we will gather extant data via geolinking with T5K participant information [25]. These include the participant’s ZIP code matched to county- and state-level data regarding, for example, STI rates, HIV incidence and prevalence, HIV viral suppression rates, residing in an Affordable Care Act Medicaid expansion state (yes/no), urban versus nonurban city/county location, state-wide PrEP policies, and pro-/anti-lesbian, gay, bisexual, and transgender policies, Human Rights Campaign state equality index [26], and the Movement Advancement Project (MAP) index [27].

### Collaboration With the *Together 5000* Study Team

T5K welcomes new collaborations. Instructions and a concept proposal form are available on our website or can be obtained by emailing the Principal Investigator (CG). Submitted concept proposals will be reviewed by CG and a core group of T5K investigators, with rapid turnaround.

## Appendix

Multimedia Appendix 1 Checklist for Reporting Results of Internet E-Surveys (CHERRIES) and Sensitivity analyses for HIV incidence estimates among *Together 5000* (T5K) study population.

## Abbreviations

CAS: condomless anal sex
CUNY: City University of New York
GBM: gay, bisexual, and other men who have sex with men
IRR: incidence rate ratio
NIH: National Institutes of Health
PrEP: pre-exposure prophylaxis
STI: sexually transmitted infection
T5K: *Together 5000*
ZIP: Zone Improvement Plan

## References

[1] (2016). Centers for Disease Control and Prevention.

[2] Horn T, Sherwood J, Remien RH, Nash D, Auerbach JD, Treatment Action Group and Foundation for Aids Research HIV Prevention Continuum Working Group (2016). Towards an integrated primary and secondary HIV prevention continuum for the United States: a cyclical process model. J Int AIDS Soc.

[3] (2014). Centers for Disease Control and Prevention.

[4] (2016). Centers for Disease Control and Prevention.

[5] (2016). New York City Department of Health and Mental Hygiene.

[6] (2016). Los Angeles County Department of Public Health.

[7] Bush S, Magnuson D, Rawlings MK, Hawkins T, McCallister S, Mera GR (2016). National AIDS Treatment Advocacy Project.

[8] Siegler AJ, Mouhanna F, Giler RM, Weiss K, Pembleton E, Guest J, Jones J, Castel A, Yeung H, Kramer M, McCallister S, Sullivan PS (2018). The prevalence of pre-exposure prophylaxis use and the pre-exposure prophylaxis-to-need ratio in the fourth quarter of 2017, United States. Ann Epidemiol.

[9] Grov C, Breslow AS, Newcomb ME, Rosenberger JG, Bauermeister JA (2014). Gay and bisexual men's use of the internet: research from the 1990s through 2013. J Sex Res.

[10] Algarin AB, Ward PJ, Christian WJ, Rudolph AE, Holloway IW, Young AM (2018). Spatial distribution of partner-seeking men who have sex with men using geosocial networking apps: epidemiologic study. J Med Internet Res.

[11] Duncan DT, Park SH, Hambrick HR, Dangerfield II DT, Goedel WC, Brewer R, Mgbako O, Lindsey J, Regan SD, Hickson DR (2018). Characterizing geosocial-networking app use among young black men who have sex with men: a multi-city cross-sectional survey in the southern United States. JMIR Mhealth Uhealth.

[12] (2016). Grants & Funding | National Institutes of Health.

[13] Grov C, Westmoreland DA, Carneiro PB, Stief M, MacCrate C, Mirzayi C, Pantalone DW, Patel VV, Nash Denis (2019). Recruiting vulnerable populations to participate in HIV prevention research: findings from the Together 5000 cohort study. Ann Epidemiol.

[14] Hull MW, Wu Z, Montaner JS (2012). Optimizing the engagement of care cascade: a critical step to maximize the impact of HIV treatment as prevention. Curr Opin HIV AIDS.

[15] Mugavero MJ, Amico KR, Horn T, Thompson MA (2013). The state of engagement in HIV care in the United States: from cascade to continuum to control. Clin Infect Dis.

[16] McCambridge J, Kypri K, Elbourne D (2014). Research participation effects: a skeleton in the methodological cupboard. J Clin Epidemiol.

[17] Adair JG (1984). The Hawthorne effect: a reconsideration of the methodological artifact. J Appl Psychol.

[18] McCambridge J, de Bruin M, Witton J (2012). The effects of demand characteristics on research participant behaviours in non-laboratory settings: a systematic review. PLoS One.

[19] Rendina HJ, Mustanski B (2018). Privacy, trust, and data sharing in web-based and mobile research: participant perspectives in a large nationwide sample of men who have sex with men in the United States. J Med Internet Res.

[20] Mbulaiteye S, Mahe C, Ruberantwari A, Whitworth J (2002). Generalizability of population-based studies on AIDS: a comparison of newly and continuously surveyed villages in rural southwest Uganda. Int J Epidemiol.

[21] Bouchet C, Guillemin F, Briançon S (1996). Nonspecific effects in longitudinal studies: impact on quality of life measures. J Clin Epidemiol.

[22] Bauermeister J, Pingel E, Zimmerman M, Couper M, Carballo-Diéguez A, Strecher VJ (2012). Data quality in web-based HIV/AIDS research: handling invalid and suspicious data. Field Methods.

[23] Khosropour CM, Johnson BA, Ricca AV, Sullivan PS (2013). Enhancing retention of an internet-based cohort study of men who have sex with men (MSM) via text messaging: randomized controlled trial. J Med Internet Res.

[24] Sullivan PS, Khosropour CM, Luisi N, Amsden M, Coggia T, Wingood GM, DiClemente RJ (2011). Bias in online recruitment and retention of racial and ethnic minority men who have sex with men. J Med Internet Res.

[25] Vaughan AS, Kramer MR, Cooper HL, Rosenberg ES, Sullivan PS (2016). Completeness and reliability of location data collected on the web: assessing the quality of self-reported locations in an internet sample of men who have sex with men. J Med Internet Res.

[26] (2015). Human Rights Campaign.

[27] (2016). Movement Advancement Project.

