# Supplementary material for: A Web-Based Study of HIV Prevention in the Era of Pre-Exposure Prophylaxis Among Vulnerable HIV-Negative Gay and Bisexual Men, Transmen, and Transwomen Who Have Sex With Men: Protocol for an Observational Cohort Study
Source: JMIR Res Protoc. 2019 Sep 17;8(9):e13715. doi: 10.2196/13715 (PMC6754683; doi:10.2196/13715)
Supplement: Multimedia Appendix 1 [file resprot_v8i8e13715_app1.pdf]

Appendix

Appendix Table 1. Sensitivity analyses for HIV incidence estimates among T5K study population in the 12- and 6-month periods prior to study enrollment

	Total	Never on PrEP	History of PrEP	RR 95% CI[a]
12 month period prior to enrollment				
12-month incidence rate (sensitivity analysis 1)[b]				
Presumed seroconversions	133	125	8.7	
Person-years at risk among seroconverters	66.7	62.3	4.3	
Person-years at risk among HIV-negative persons	4814	4101	713	
Incidence rate per 100 person years (95% CI)	2.73 (2.30-3.23)	2.99 (2.52-3.58)	1.21 (0.62-2.32)	2.48 (1.26-5.0)
12-month incidence rate (sensitivity analysis 2) [c]				
Presumed seroconversions	135	127	8.7	
Person-years at risk among seroconverters	67.7	63.4	4.334	
Person-years at risk among HIV-negative persons	4814	4101	713	
Incidence rate per 100 person years (95% CI)	2.77 (2.34-3.27)	3.04 (2.56-3.63)	1.21 (0.62-2.32)	2.52 (1.28-5.07)
12-month incidence rate (sensitivity analysis 3) [d]				
Presumed seroconversions	196	186	10	
Person-years at risk among seroconverters	98	93	5	
Person-years at risk among HIV-negative persons	4814	4101	713	
Incidence rate per 100 person years (95% CI)	3.99 (3.45-4.57)	4.44 (3.82-5.10)	1.39 (0.71-2.50)	3.18 (1.74-6.30)
6 months prior to enrollment				
6-month incidence rate (sensitivity analysis 4) [e]				
Presumed recent seroconversions	84	77	6.3	
Person-years at risk among seroconverters	20.9	19.3	1.6	
Person-years at risk among HIV-negative persons	2407	2051	357	
Incidence rate per 100 person years (95% CI)	3.44 (2.79-4.28)	3.73 (2.97-4.64)	1.77 (0.97-3.28)	2.11 (1.02-5.61)
6-month incidence rate (sensitivity analysis 5) [f]				

Presumed recent seroconversions	91	85	6.3	
Person-years at risk among seroconverters	22.85	21.26	1.58	
Person-years at risk among HIV-negative persons	2407	2051	357	
Incidence rate per 100 person years (95% CI)	3.76 (3.04-4.59)	4.11 (3.31-5.06)	1.77 (0.97-3.28)	2.32 (1.13-6.17)

[a] mid P exact test

[b] Sensitivity analysis 1 builds on the base 12 month estimate and further assumes that the last HIV-negative test was 18 months ago for those reporting it was >12 months ago, with timing of seroconversion distributed evenly between enrollment and 18 months prior

[c] Sensitivity analysis 2 builds on the Sensitivity analysis 1 and further assumes that 2/3 of those with no prior HIV test serconverted during the 12 months prior to enrollment

[d] Sensitivity analysis 3 assumes that all 195 persons who were identified as HIV+ at enrollment seroconverted during the 12 months prior to enrollment

[e] Sensitivity analysis 4 builds on thebase 6-month estimate and further assumes that the last HIV-negative test was 18 months ago for those reporting it was >12 months ago, with timing of seroconversion distributed evenly between enrollment and 18 months prior

[f] Sensitivity analysis 5 builds on Sensitivity analysis 4 and further assumes that half of those with no prior HIV test seroconverted during the 12 months prior to enrollment

Supplemental Table 1. Identifying statements that address different aspects of the Checklist for Reporting Results of Internet E-Surveys (CHERRIES), *Together 5000* Study, 2017-2018.

Checklist for Reporting Results of Internet E-Surveys (CHERRIES)

Checklist for Reporting Results of Internet E-Surveys (CHERRIES)

Item Category	*Checklist Item*	*Explanation*	*Pg*	*Line*	*Quote*
**Design**					
	Describe survey design	Describe target population, sample frame. Is the sample a convenience sample? (In "open" surveys this is most likely.)	5	94	"Internet-based strategies to enroll a large sample of HIV-negative men, transmen, and transwomen who have sex with men GBM aged 16 to 49 at high risk of HIV acquisition."
**IRB (Institutional Review Board) approval and informed consent process**					
	IRB approval	Mention whether the study has been approved by an IRB.	10	206	"The T5K study protocol was approved by the Institutional Review Board of the City University of New York."

	Informed consent	Describe the informed consent process. Where were the participants told the length of time of the survey, which data were stored and where and for how long, who the investigator was, and the purpose of the study?	6	114	"Potential participants were directed to a secure enrollment survey in their device's web browser, and presented with a screen describing study participation and eliciting informed consent. The informed consent described the incentive schedule: $15 for completing a secondary survey (i.e., one after the enrollment survey) if they were eligible, and another $15 for completing self-administered at-home HIV testing (i.e., oral fluid sample returned to the study laboratory for testing). Additional incentives, described in the informed consent, are available to participants who complete prospective longitudinal follow up assessments."
	Data protection	If any personal information was collected or stored, describe what mechanisms were used to protect unauthorized access.	7	144	"All participants were assigned a unique identifier at study enrollment and this unique identifier was used for all study databases and datasets. Participant contact information were stored in an encrypted database separated from participant questionnaire answers and other study-related information. Only study staff were allowed access to study databases."
**Development and pre-testing**					
	Development and testing	State how the survey was developed, including whether the usability and technical functionality of the electronic questionnaire had been tested before fielding the questionnaire.	7	129	"The survey was programmed into Qualtrics survey software and tested by project staff. Measures had been previously used by members of the research team or were derived from published research." "Prior to activating the survey, members of the project staff tested the survey extensively for usability and technical function. The survey was tested on Windows, Mac, iOS, and Android devices and on Chrome, Firefox, Internet Explorer, Opera, and Safari web browsers."
**Recruitment process and description of the sample having access to the questionnaire**					
	Open survey versus closed survey	An "open survey" is a survey open for each visitor of a site, while a closed survey is only open to a sample which			Open-enrollment and closed follow-up noted throughout sections.

		the investigator knows (password-protected survey).			
	Contact mode	Indicate whether or not the initial contact with the potential participants was made on the Internet. (Investigators may also send out questionnaires by mail and allow for Web-based data entry.)	7	127	"Interested potential participantsindividuals were screened for eligibility via an online survey collecting data on sexual behavior, substance use, demographic characteristics, and history of PrEP/PEP use."
	Advertising the survey	How/where was the survey announced or advertised? Some examples are offline media (newspapers), or online (mailing lists – If yes, which ones?) or banner ads (Where were these banner ads posted and what did they look like?). It is important to know the wording of the announcement as it will heavily influence who chooses to participate. Ideally the survey announcement should be published as an appendix.	6	105	"Participants were recruited via ads on men-for-men geosocial sexual networking smartphone apps (Figure 1)."
**Survey administration**					
	Web/E-mail	State the type of e-survey (eg, one posted on a Web site, or one sent out through e-mail). If it is an e-mail survey, were the responses entered manually into a database, or was there an automatic method for capturing responses?	7	127	"Interested potential participantsindividuals were screened for eligibility via an online survey collecting data on sexual behavior, substance use, demographic characteristics, and history of PrEP/PEP use."
	Context	Describe the Web site (for mailing list/newsgroup) in which the survey was posted. What is the Web site about, who is visiting it, what are visitors normally looking for? Discuss to what degree the content of the Web site could pre-select the sample or influence the results. For example, a survey about vaccination on a anti-immunization Web site will have different results from a Web survey conducted on a government Web site	6	105	"Participants were recruited via ads on men-for-men geosocial sexual networking smartphone apps (Figure 1)."
	Mandatory/voluntary	Was it a mandatory survey to be filled in by every visitor who wanted to enter the Web site, or was it a voluntary survey?			N/A

Incentives	Were any incentives offered (eg, monetary, prizes, or non-monetary incentives such as an offer to provide the survey results)?	6	117	"The informed consent described the incentive schedule: $15 for completing a secondary survey (i.e., one after the enrollment survey) if they were eligible, and another $15 for completing self-administered at-home HIV testing (i.e., oral fluid sample returned to the study laboratory for testing). Additional incentives, described in the informed consent, are available to participants who complete prospective longitudinal follow up assessments."
Time/Date	In what timeframe were the data collected?	6	103	"Enrollment for T5K began in October 2017 and concluded in June 2018, when 67,166 of the estimated 649,000 (10.3%) males eligible for PrEP across the U.S. were using it."
Randomization of items or questionnaires	To prevent biases items can be randomized or alternated.	7	133	"Survey items were not randomized by participant."
Adaptive questioning	Use adaptive questioning (certain items, or only conditionally displayed based on responses to other items) to reduce number and complexity of the questions.	7	131	"Our online survey was divided into thematic blocks based on question content and used adaptive questions based on survey responses from the participants. Examples include survey questions about known HIV status, PrEP use, and main sexual partners."
Number of Items	What was the number of questionnaire items per page? The number of items is an important factor for the completion rate.	7	134	"Because of the skip and/or display logic used within the survey, the number of items answered by participants and displayed per page of the web-survey varied by subject and participant responses."
Number of screens (pages)	Over how many pages was the questionnaire distributed? The number of items is an important factor for the completion rate.	7	136	"However, to improve ease of use on mobile devices and reduce survey fatigue, each page contained 1-2 questions."
Completeness check	It is technically possible to do consistency or completeness checks before the questionnaire is submitted. Was this done, and if "yes", how (usually JAVAScript)? An alternative is to check for completeness after the questionnaire has been submitted (and highlight mandatory items). If this has been done, it should be reported. All items should provide a non-response option such as "not applicable" or "rather not say", and selection of one response option should be enforced.			N/A

	Review step	State whether respondents were able to review and change their answers (eg, through a Back button or a Review step which displays a summary of the responses and asks the respondents if they are correct).	7	137	"Again, due to skip and/or display logic depending on previous responses, participants could not click back to view or change a previous response. If a participant chose an incorrect response, they could contact the study to reset that response."
**Response rates**					
	Unique site visitor	If you provide view rates or participation rates, you need to define how you determined a unique visitor. There are different techniques available, based on IP addresses or cookies or both.	8	152	"We followed established and effective measures to minimize repeat participation and fraudulent manipulation of HIV testing procedures.[11] Our enrollment survey blocked multiple submissions, our databases flagged duplicate contact information, and all mailing addresses were validated with the U.S. Postal ServiceDuplicate individuals were identified and removed using IP addresses and XYZ. We also assessed time to completion of our online surveys and checked for variability in response sets."
	View rate (Ratio of unique survey visitors/unique site visitors)	Requires counting unique visitors to the first page of the survey, divided by the number of unique site visitors (not page views!). It is not unusual to have view rates of less than 0.1 % if the survey is voluntary.	10	210	"In total, 43,161 individuals began our enrollment survey and 22,091 (51.2%) completed it (Figure 2). Of non-completers (n = 21,070), 61% (n = 12,862) closed their browser window on the informed consent page (i.e., immediately)."
	Participation rate (Ratio of unique visitors who agreed to participate/unique first survey page visitors)	Count the unique number of people who filled in the first survey page (or agreed to participate, for example by checking a checkbox), divided by visitors who visit the first page of the survey (or the informed consents page, if present). This can also be called “recruitment” rate.	10-12	212-229	"Of completers, 9,193 (41.6%) were eligible; however, 1,023 were excluded because we determined their response to be a duplicate entry. Of the remaining 8,807 participants who provided informed consent to participate in the study, 30 were excluded as they did not provide contact information. Thus, the final sample was 8,777 consented participants from all 50 U.S. states, Puerto Rico and Guam (Figure 3)."
	Completion rate (Ratio of users who finished the survey/users who agreed to participate)	The number of people submitting the last questionnaire page, divided by the number of people who agreed to participate (or submitted the first survey page). This is only relevant if there is a separate “informed consent” page or if the survey goes over several pages. This is a measure for attrition. Note that “completion” can involve leaving questionnaire items blank. This is not a measure for how completely questionnaires were filled in. (If you need a measure for this, use the word “completeness rate”.)	10-12	212-229	"Of completers, 9,193 (41.6%) were eligible; however, 1,023 were excluded because we determined their response to be a duplicate entry. Of the remaining 8,807 participants who provided informed consent to participate in the study, 30 were excluded as they did not provide contact information. Thus, the final sample was 8,777 consented participants from all 50 U.S. states, Puerto Rico and Guam (Figure 3)."

**Preventing multiple entries from the same individual**					
	Cookies used	Indicate whether cookies were used to assign a unique user identifier to each client computer. If so, mention the page on which the cookie was set and read, and how long the cookie was valid. Were duplicate entries avoided by preventing users access to the survey twice; or were duplicate database entries having the same user ID eliminated before analysis? In the latter case, which entries were kept for analysis (eg, the first entry or the most recent)?	8	152	"We followed established and effective measures to minimize repeat participation and fraudulent manipulation of HIV testing procedures including recording IP addresses of participants and using cookies to block repeated attempts"
	IP check	Indicate whether the IP address of the client computer was used to identify potential duplicate entries from the same user. If so, mention the period of time for which no two entries from the same IP address were allowed (eg, 24 hours). Were duplicate entries avoided by preventing users with the same IP address access to the survey twice; or were duplicate database entries having the same IP address within a given period of time eliminated before analysis? If the latter, which entries were kept for analysis (eg, the first entry or the most recent)?	8	152	"We followed established and effective measures to minimize repeat participation and fraudulent manipulation of HIV testing procedures including recording IP addresses of participants and using cookies to block repeated attempts"
	Log file analysis	Indicate whether other techniques to analyze the log file for identification of multiple entries were used. If so, please describe.	8	157	"Multiple entries were identified by email addresses and/or phone numbers. Additionally, the data manager manually checked for duplicate entries during baseline data collection."
	Registration	In “closed” (non-open) surveys, users need to login first and it is easier to prevent duplicate entries from the same user. Describe how this was done. For example, was the survey never displayed a second time once the user had filled it in, or was the username stored together with the survey results and later eliminated? If the latter, which entries were kept for analysis (eg, the first entry or the most recent)?			N/A
**Analysis**					
	Handling of incomplete questionnaires	Were only completed questionnaires analyzed? Were questionnaires which terminated early (where, for example, users did not go through all questionnaire pages) also analyzed?	10	220	"The following descriptive statistics of the cohort and HIV incidence rates calculated for this profile included responses from enrolled participants meeting eligibility criteria and completed questionnaires."

	Questionnaires submitted with an atypical timestamp	Some investigators may measure the time people needed to fill in a questionnaire and exclude questionnaires that were submitted too soon. Specify the timeframe that was used as a cut-off point, and describe how this point was determined.	8	160	"We also assessed time to completion of our online surveys and checked for variability in response sets."
	Statistical correction	Indicate whether any methods such as weighting of items or propensity scores have been used to adjust for the non-representative sample; if so, please describe the methods.			N/A
